# Organ Diseases of Women

**Published:** 1897-07

**Authors:** 


### BOOK NOTICE.

ORGAN DISEASES OF WOMEN; NOTABLY, ENLARGEMENTS AND DISPLACEMENTS OF THE UTERUS AND STERILITY, CONSIDERED AS CURABLE BY MEDICINES. By J. Compton Burnett, M. D., author of "Tumors of the Breast," etc. Philadelphia, Boericke & Tafel, 1897. Price, cloth, $1.00; by mail, $1.05.

The author of this small volume is well known to the homeopathic profession by his numerous monographs on various medical subjects, mainly, as showing the curability of certain diseases hitherto regarded as totally incurable, or else as amenable only to operation.

The cases given in these different volumes show Dr. Burnett to be a very remarkable physician even for a homeopathist. He certainly accomplishes results that few other men attain. The volume now before us increases, if anything, the lustre of his achievements, and gives strong encouragement to the beginner in the new school of practice to emulate him.

He denounces the pessary, shows its uselessness, and demonstrates the proper way of treating conditions for which the pessary is usually prescribed, and gives the names of the best remedies for it.

He emphasizes his own method of treatment by citing a case of prolapsus cured by a clergyman, who was an amateur homeopathist, with Sepia. He laughs at allopathic opposition and skepticism, and declares that "The opinion of allopaths on the value of Homeopathy are nothing but spiteful splutter, vulgar and nasty."

After the delivery of this opinion he proceeds to the narration of triumphant results of homeopathic treatment of barren women, who thereafter had children, as desired.

In these cases he gives Cedron where mental excitement seems to bring on the menses, and with it the patient complains of coldness in the abdomen. This is the keynote, apparently, for Cedron.

Vigorous denunciations of the subterfuges resorted to for the prevention of conception are indulged, and the consequences, especially enlargement of the uterus, pointed out. In these cases of enlargement of the uterus, if he finds enlargement of the spleen, he gives Urtica-urens.

For enlargement of the uterus his principal remedies are Bellis-perennis and Fraxinus-Americanus, and they certainly give marvelous results. He admits that his giving of these medicines in these cases is mostly on the principles of Organopathy, and then he defends Organopathy, and claims it to be a valuable appendix to Hahnemann's teachings. His directions for the application of Organopathy are thus stated: "Where the organ-ailing is primary to the organ, use organ remedies, in little material doses, frequently repeated; where the organ-ailing is of a piece pathologically with that of the organism, use the homeopathic simillimum in potency, infrequently repeated." "That is how I work," he adds, "with much satisfaction and delight at the curative results so obtained."

The author's favorite remedy for morning sickness is Medorrhinum. "No case of severe vomiting in pregnancy," he says, "should be given up as hopeless unless Medorrhinum c. and cc. in very infrequent dose has been tried. In my experiences no other *one* remedy meets such a large percentage of these cases curatively."

Tritricum-repens is his great remedy for painful urination. Injections into the vagina he denounces, and declares that no constitutional diseases of the womb and ovaries are ever cured by operations.

In view of his remarkable clinical results, he is quite strongly fortified in the position he takes, in which he will be warmly indorsed by every sincere homeopathist.

With this short statement of some of the salient points of the little volume, we commend the book to the careful perusal of the reader, with the suggestion that if he have similar cases he may find in its pages a helpful suggestion.

